# Effects of feeding type on gut microbiota and atopic dermatitis in cesarean delivered infants: a combined birth cohort study

**DOI:** 10.1007/s44463-025-00040-x

**Published:** 2026-02-11

**Authors:** Minyoung Jung, Sukyung Kim, Jeongmin Song, Hyun Mi Kim, Yeonghee Kim, Min Hee Lee, Yechan Kyung, Minji Kim, Sanghee Shin, Sehun Jang, Woong Bom Kim, Hyeonwoo Kim, Bomin Koh, Bongsang Kim, Junghyun Lim, Chanwoo Kim, Jaeho Lee, Suk-Joo Choi, Soo-Young Oh, Jihyun Kim, Kangmo Ahn

**Affiliations:** 1 Department of Pediatrics, Samsung Medical Center, Sungkyunkwan University School of Medicine, Seoul, Republic of Korea; 2 Department of Pediatrics, Sungkyunkwan University School of Medicine, Changwon, Republic of Korea; 3 Department of Pediatrics, Chungnam National University Sejong Hospital, Chungnam National University College of Medicine, Sejong, Republic of Korea; 4 Department of Pediatrics, Chung-Ang University Hospital, Chung-Ang University College of Medicine, Seoul, Republic of Korea; 5 Department of Pediatrics, Seoul National University Bundang Hospital, Seongnam, Republic of Korea; 6 eGnome, Inc, Seoul, Republic of Korea; 7 Department of Agricultural Biotechnology and Research Institute of Agriculture and Life Sciences, Seoul National University, Seoul, Republic of Korea; 8 Division of Beverage, Lotte R&D Center, Seoul, Republic of Korea; 9 Department of Obstetrics and Gynecology, Samsung Medical Center, Sungkyunkwan University School of Medicine, Seoul, Republic of Korea; 10 Department of Health Sciences and Technology, Samsung Advanced Institute for Health Sciences & Technology, Seoul, Republic of Korea

**Keywords:** Atopic dermatitis, Bifidobacterium, Cesarean section, Gut microbiota, Probiotic formulas

## Abstract

To investigate the effect of early feeding type on gut microbiota composition and atopic dermatitis (AD) development in cesarean-delivered infants, and to evaluate whether probiotic-supplemented formula can mitigate microbial and immunologic disadvantages associated with cesarean delivery. A total of 129 infants delivered by cesarean section were enrolled and classified into three groups: Group A (exclusively breastfed), Group B (fed standard formula), and Group C (fed probiotic-fortified formula). Stool samples were collected at 6–8 weeks of age and analyzed using 16S rRNA gene sequencing. Microbial diversity indices and taxonomic composition were compared, and the incidence of AD by 6 months was assessed. The incidence of AD by 6 months was 8.3% in Group A, 66.7% in Group B, and 25.0% in Group C (*p* = 0.793). Group A showed the highest gut microbial diversity (Shannon, Chao1, and observed amplicon sequence variants), with significantly lower diversity in Group C (adjusted *p* < 0.017). No significant differences were observed in the Simpson index. Group C exhibited a significantly higher relative abundance of *Bifidobacterium* compared with Groups A and B (adjusted *p* < 0.001), while *Lactobacillus* was most abundant in Group A, with significant differences across all groups (adjusted *p* < 0.001). The incidence of AD did not significantly differ between Group A and Group C . While early probiotic supplementation via infant formula may support beneficial bacterial colonization in cesarean-delivered infants, its potential role in AD prevention remains to be clarified through further longitudinal studies.

## Introduction

Atopic dermatitis (AD) is the most common chronic inflammatory skin disease with increasing prevalence in Korean children and places a considerable burden on the healthcare system and quality of life (Lee et al., 2022; Shin et al., 2024). In addition to genetic susceptibility, environmental factors such as dietary patterns, air pollution, and early antibiotic exposure have been implicated in AD pathogenesis (Choi et al., 2025; Weidinger & Novak, 2016). Recent evidence suggests that the gut microbiome also plays a crucial role in immune system development and regulation, with microbial communities and their metabolites contributing to immune homeostasis and modulating susceptibility to immune-mediated diseases (Pessoa et al., 2023; Rooks & Garrett, 2016). The concept of the gut-skin axis highlights the effects of gut microbial composition and the modulation of its metabolite on systemic immune responses and skin inflammation (Lee et al., 2018).

Infants who develop extrinsic AD by 2 years of age showed lower gut microbial diversity at 1 month, particularly with a reduction in the phylum Bacteroidetes and genus *Bacteroides* (Abrahamsson et al., 2012). In addition, infants with AD displayed a distinct gut microbiota pattern, with elevated levels of proteolytic bacteria such as *Enterobacter*, *Klebsiella*, and *Escherichia coli* and reduced levels of beneficial genera including *Enterococcus*,* Lactobacillus*, and *Bifidobacterium* (Pantazi et al., 2023). These dysbiotic signatures precede clinical onset, indicating a critical early-life window, in which gut microbiota can influence AD development through immune education.

Perinatal factors, such as mode of delivery and early feeding practices, are known to shape the composition of the early gut microbiome (Jeong, 2022; Madan et al., 2016). A systematic review demonstrated that infants delivered by cesarean section showed reduced colonization by beneficial phyla such as Actinobacteria and Bacteroidetes within the first 3 months of life, whereas those delivered vaginally had higher abundances of *Bifidobacterium* and *Bacteroides* (Rutayisire et al., 2016). Similarly, in a meta-analysis, non-exclusively breastfed infants had consistently higher relative abundances of Bacteroidetes and Firmicutes compared with infants exclusively breastfed, with even greater differences observed in formula-fed infants (Ho et al., 2018). Although breastfed infants show lower overall microbial diversity, they exhibit higher levels of beneficial bacteria such as *Bifidobacterium* and *Lactobacillus*, along with reduced abundances of pro-inflammatory taxa such as *Proteobacteria* and *Clostridium* (Davis et al., 2022a).

Growing evidence highlights the impact of maternal gut microbiota on the infant’s immune and microbial development. Du et al. showed that higher maternal microbial α-diversity and enrichment of short-chain fatty acid-producing bacteria such as *Faecalibacterium* and *Roseburia,* as well as elevated levels of linoleic acid derivatives and flavonoids were associated with a lower risk of infant AD (Du et al., 2025). These findings suggest that maternal microbial composition and its metabolites during pregnancy may help shape the early-life gut environment and influence immune tolerance in offspring.

Although these factors have been extensively studied, few investigations have addressed whether probiotic fortification can compensate for gut microbial alterations associated with cesarean delivery, particularly in high-risk infants. To address this gap, we conducted a combined analysis of three Korean birth cohorts, focusing on infants delivered by cesarean section. This study aimed to evaluate whether early feeding type, including exclusive breastfeeding, standard formula, and probiotic-fortified formula, modulates gut microbiota composition and influences the development of AD in cesarean-delivered infants.

## Materials and methods

### Study population and design

This study analyzed data from three prospective birth cohorts conducted in Korea, each designed to investigate early-life risk factors for allergic diseases. The first cohort recruited pregnant women from the general population without specific inclusion criteria. The second cohort stratified participants into high-risk and control groups based on the allergic disease history and parental sensitization status. At enrollment, both parents underwent skin prick testing (SPT) with common 8 inhalant allergens (Cho et al., 2024; Lee et al., 2022). Infants were classified as high-risk if either (1) at least one parent had both a positive SPT result and a history of asthma or allergic rhinitis, or (2) at least one parent or sibling had physician-diagnosed AD. The control group was defined by the absence of allergic disease history and negative SPT results in both parents. The third cohort included only women who delivered via cesarean section.

All cohorts enrolled pregnant women during their third trimester, and infants were followed from birth to 6 months of age. Inclusion criteria for the present analysis were (1) cesarean delivery, (2) availability of stool samples collected at 1–2 months of age, (3) completion of clinical follow-up by 6 months, and (4) presence of a family history of allergic diseases. At enrollment, parents completed a questionnaire regarding basic demographic information and family history of allergic diseases. Each original cohort study was approved by the respective institutional review boards (IRBs) at the time of data collection, and the current secondary analysis was approved by the IRB of Samsung Medical Center (IRB No. SMC-2025-04-013). Written informed consent was obtained from the legal guardians of all participants during original cohort enrollment.

## Clinical data collection

Infants delivered via cesarean section were categorized into three groups based on feeding type. Group A included exclusively breastfed infants. Group B comprised those fed with standard commercial formula. Infants in Group C received a commercially available probiotic-fortified formula including *B. longum* KACC 91,563 (1.1 × 10⁸–1.0 × 10⁹ colony-forming units [CFU]), *L. rhamnosus* GG (7.5 × 10⁷ CFU), *L. reuteri* (2.5 × 10⁶ CFU), and *B. lactis* BB-12 (1.1 × 10⁸ CFU). The formula was supplied free of charge by Lotte Wellfood Co., Ltd. (Seoul, Korea), which had no involvement in the study design, data collection, data analysis, interpretation of results, or manuscript preparation. Infants in Group C did not receive exclusive breastfeeding. Demographic and perinatal factors were collected, including birth weight, gestational age, antibiotic exposure, additional probiotic supplementation, and household environmental characteristics. Infants who received antibiotic treatment within the first two months were excluded from the analysis.

Scheduled clinical visits were conducted at 1–2 months and 6 months of age. At each visit, pediatric allergists performed a clinical examination to diagnose AD based on the Hanifin and Rajka criteria (Kulthanan et al., 2021). In cases where hospital visits were not feasible, parents completed a structured questionnaire regarding any physician-diagnosed AD. When the parents reported any suspicious symptoms, infants were evaluated at outpatient clinics.

## Stool sample collection and gut microbiota profiling

Stool samples were collected at 6–8 weeks of age. Fecal samples were collected by caregivers using a stool collection kit capable of preserving at room temperature for up to 8 weeks. Once the study team was notified, samples were promptly delivered to the research facility via same- or next-day courier service and immediately stored at − 80 °C upon arrival. Microbial profiling was conducted using two distinct approaches depending on the cohort. For a subset of participants, the hypervariable V3–V4 regions of the 16S rRNA gene were amplified and sequenced on an Illumina MiSeq platform (Illumina, San Diego, CA, USA). The resulting 16S rRNA gene sequence data were processed using Quantitative Insights Into Microbial Ecology (QIIME) software (v1.9.1) (Caporaso et al., 2010).

Using high-quality sequences (Phred ≥ Q20), operational taxonomic units (OTUs) were assigned with the open-reference method, mapping sequences with ≥ 97% identity to known sequences in the Greengenes database (v13.8) using UCLUST alignment algorithms, supplemented with taxonomic annotation from the EzBioCloud database (http://www.ezbiocloud.net) (DeSantis et al., 2006; Edgar, 2010; Yoon et al., 2017).

For the remaining samples, the full-length 16S–ITS–23S rRNA operon region was amplified and sequenced using the Oxford Nanopore Technologies (ONT) MinION platform. Raw reads were demultiplexed and trimmed to remove adapter sequences using Porechop (v0.2.4), followed by read filtering with NanoFilt (v2.8.0), retaining only reads with an average Phred quality score ≥ 14 and lengths consistent with the reference sequence range of the curated database used by MIrROR (v1.0) (Yoon et al., 2017). Taxonomic classification was then performed using MIrROR, which aligns full-length 16S–ITS–23 S rRNA reads against the database and assigns taxonomy based on sequence similarity.

### Statistical analysis

Clinical characteristics were compared among the three groups (Group A, Group B, and Group C). Categorical variables are presented as numbers and percentages, and continuous variables as medians with interquartile ranges (IQRs). Categorical variables, including the incidence of AD by 6 months of age, were analyzed using the Chi-square test. Continuous variables, such as alpha diversity indices and the relative abundances of bacterial taxa at the phylum and genus levels, were compared using the Kruskal–Wallis test. For multiple group comparisons with a significant Kruskal–Wallis test result, pairwise comparisons were performed using Bonferroni-adjusted post hoc tests. All statistical analyses were performed using GraphPad Prism (version 10.4.2; GraphPad Software, San Diego, CA, USA), and Stata (version 17.0; StataCorp LLC, College Station, TX, USA). A two-tailed *P* value < 0.05 was considered statistically significant.

## Results

### Study population and AD development

A total of 129 infants were included in the analysis, comprising 12 in the exclusively breastfed group (Group A), 94 in the standard formula-fed group (Group B), and 23 in the probiotic-fortified formula-fed group (Group C). No significant differences were observed among the groups in sex, gestational age, birth weight, family history of allergic diseases, maternal educational level, household mold, and tobacco smoke exposure (Table 1).

**Table 1:** Characteristics of study participants

Variables	Group A (*n* = 12)	Group B (*n* = 94)	Group C (*n* = 23)	*p*
Male, n (%)	9 (75.0)	54 (57.5)	16 (69.6)	0.333
Birth weight, kg	3.2 (3.0–3.4)	3.2 (2.9–3.5)	3.2 (2.9–3.5)	0.943
Gestational age	39 wks + 1 d (38wks + 3d − 40wks + 1d)	38 wks + 5 d (38wks + d – 39wks + 1d)	38 wks + 5 d (38wks – 39wks + 1d)	0.364
Family history of allergic disease﻿, n (%)	6 (50.0)	63 (67.0)	12 (52.2)	0.263
Household exposure to smoke, n (%)	3 (30.0)	12 (29.3)	5 (21.7)	0.788
Visible mold exposure﻿, n (%)	3 (30.0)	8 (19.5)	6 (26.1)	0.918
Mother’s educational level (*n* = 74)				0.432
High school﻿, n (%)	0 (0)	0 (0)	1 (4.4)	
College﻿, n (%)	1 (10.0)	5 (12.2)	3 (13.0)	
University﻿, n (%)	6 (60.0)	21 (51.2)	16 (69.6)	
Graduate school﻿, n (%)	3 (30.0)	15 (36.6)	3 (13.0)	
Probiotics use﻿, n (%)	7 (58.3)	56 (60.2)	13 (56.5)	0.946

**Group A**, infants delivered by cesarean section and exclusively breastfed; **Group B**, infants delivered by cesarean section and fed with standard formula; **Group C**, infants delivered by cesarean section and fed with probiotic-fortified formula

**Fig. 1:**
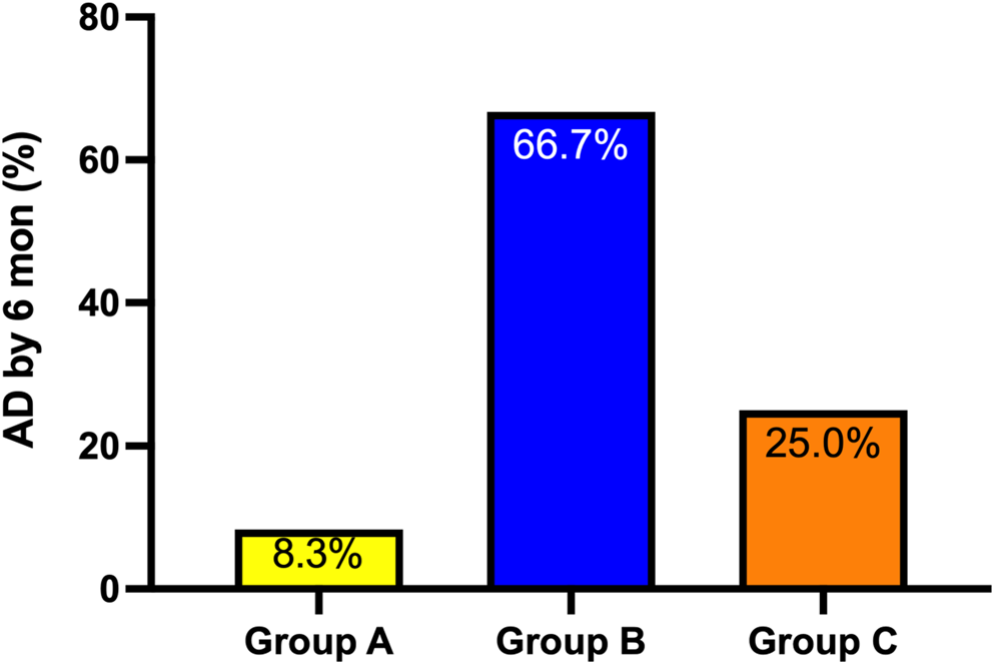
Incidence of atopic dermatitis (AD) by 6 months of age across feeding groups. **Group A**, infants delivered by cesarean section and exclusively breastfed; **Group B**, infants delivered by cesarean section and fed with standard formula; **Group C**, infants delivered by cesarean section and fed with probiotic-fortified formula

By 6 months of age, the incidence of AD was 8.3% in Group A, 66.7% in Group B, and 25.0% in Group C, but the difference was not statistically significant (*p* = 0.793) (Fig. 1).

## Gut Microbiome diversity at 2 months of age

**Fig. 2:**
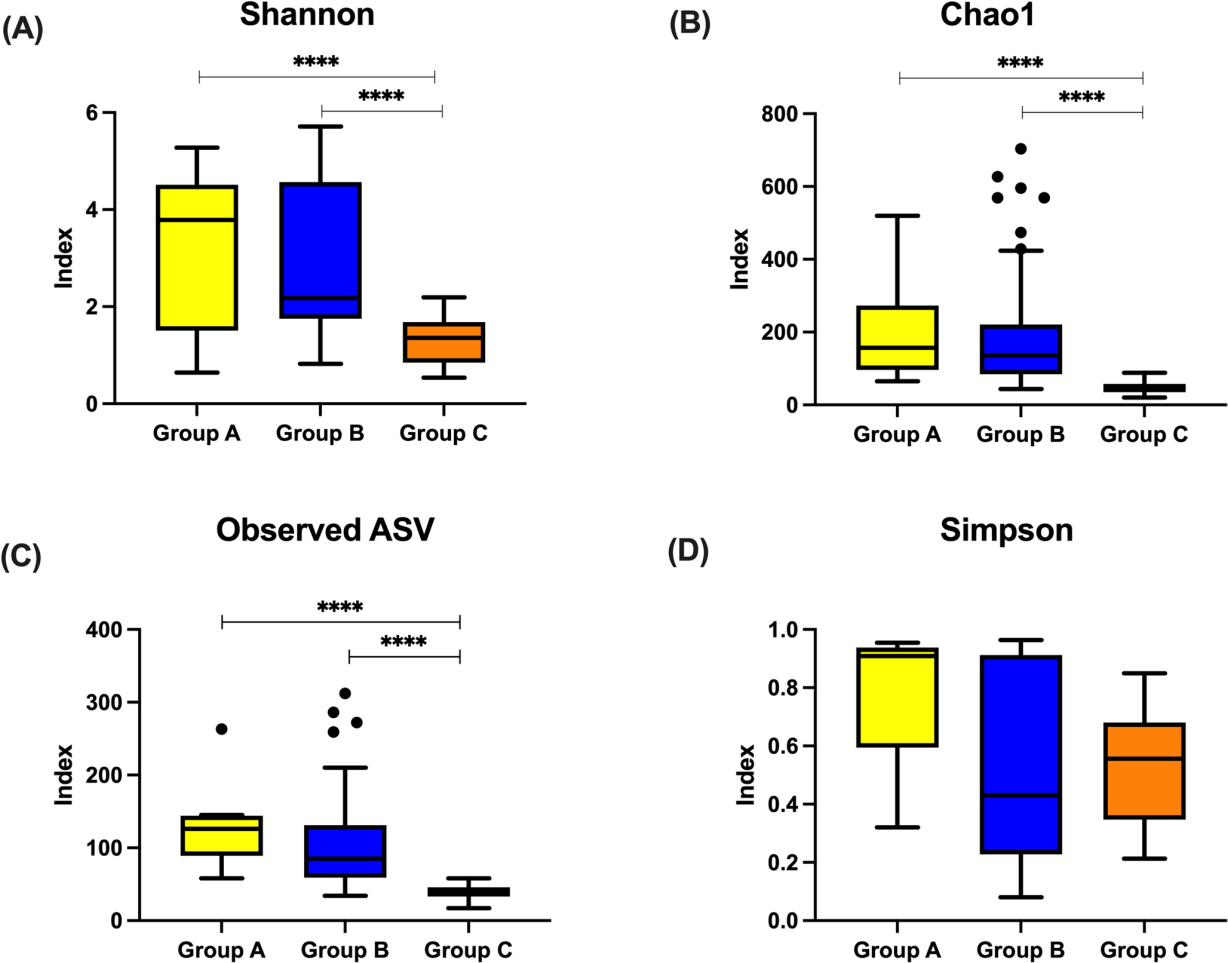
Alpha diversity of gut microbiota at 2 months of age across feeding groups. Statistical analysis was performed using the Kruskal–Wallis test with Bonferroni-corrected post hoc comparisons. ****p < 0.0001 (Bonferroni-adjusted). (**A**) Shannon diversity index, (**B**) Chao1 richness index, (**C**) Simpson diversity index (**D**) observed amplicon sequence variants (ASVs) **Group A**, infants delivered by cesarean section and exclusively breastfed; **Group B**, infants delivered by cesarean section and fed with standard formula; **Group C**, infants delivered by cesarean section and fed with probiotic-fortified formula

The Shannon diversity index was highest in Group A (median 3.78, IQR 1.51–4.51), followed by Group B (median 2.18, IQR 1.75–4.57) and Group C (median 1.36, IQR 0.85–1.68) (*p* < 0.001) (Fig. 2A). Similar trends were observed in the Chao1 richness and observed amplicon sequence variants (ASVs) (Fig. 2B and C). Bonferroni-adjusted pairwise comparisons demonstrated significant differences between Group A and C, and between Group B and C (adjusted *p* < 0.017), but not between Group A and B. There was no significant difference in the Simpson index among the three groups (Fig. 2D).

## Taxonomic composition of the gut microbiota at 2 months of age

**Fig. 3:**
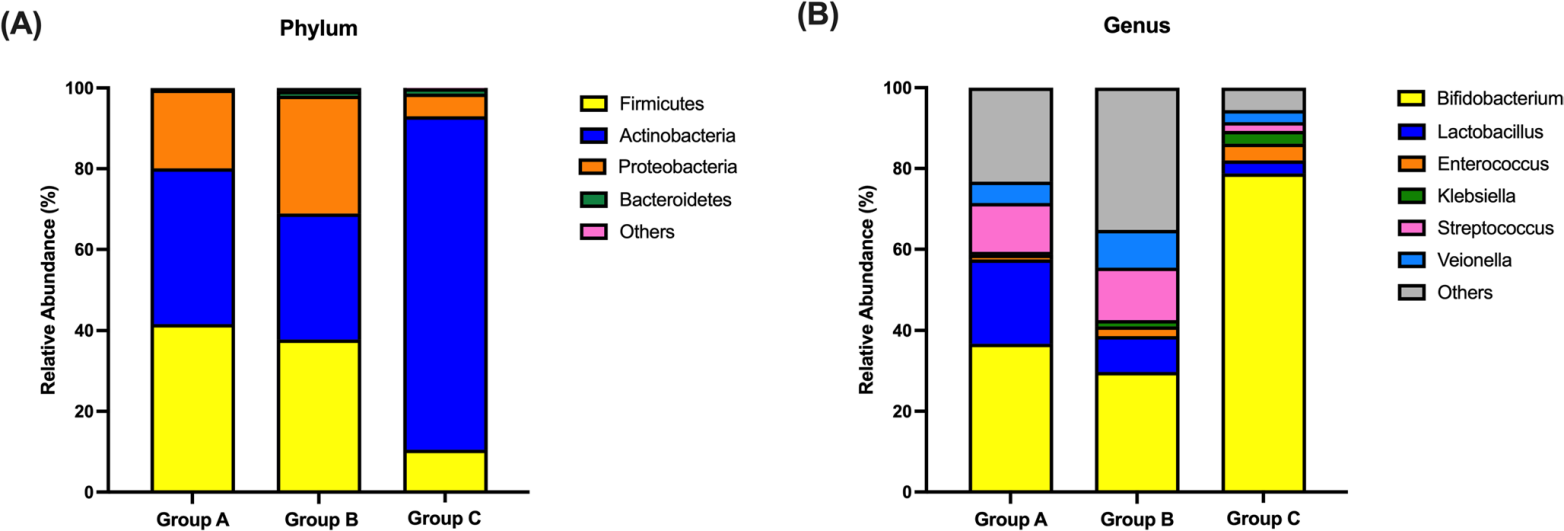
Composition of gut microbiota at 2 months of age across feeding groups. (**A**) Relative abundance of bacterial taxa at the phylum level. (**B**) Relative abundance at the genus level. **Group A**, infants delivered by cesarean section and exclusively breastfed; **Group B**, infants delivered by cesarean section and fed with standard formula; **Group C**, infants delivered by cesarean section and fed with probiotic-fortified formula

At the phylum level, Group C showed marked dominance of *Actinobacteria* (82.5%), followed by *Firmicutes* (10.5%), *Proteobacteria* (5.6%), and *Bacteroidetes* (1.3%) (Fig. 3A). In contrast, Group A and Group B exhibited a more even distribution. Group A consisted of 41.6% *Firmicutes*, 38.5% *Actinobacteria*, and 19.5% *Proteobacteria*, while Group B included 37.8% *Firmicutes*, 31.1% *Actinobacteria*, and 29.2% *Proteobacteria*.

At the genus level, *Bifidobacterium* was most abundant in Group C, compared to Group A and Group B (Fig. 3B). *Lactobacillus* and *Streptococcus* were more abundant in Group A. Additionally, *Streptococcus* abundance was low in Group C despite comparable levels in Group A and Group B.

### Differential abundance of bacterial genera by feeding type

**Fig. 4:**
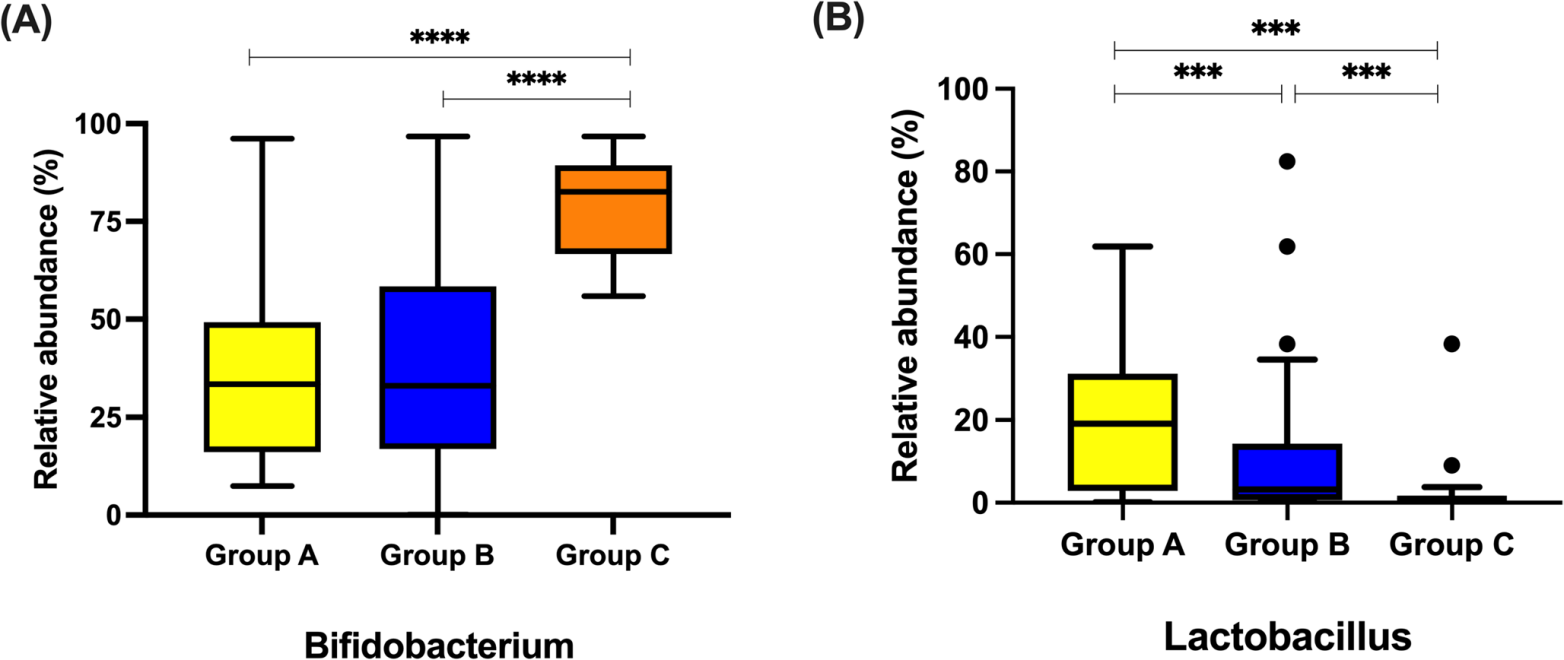
Comparison of selected bacterial genera at 2 months of age across feeding groups. Statistical analysis was performed using the Kruskal–Wallis test with Bonferroni-corrected post hoc comparisons. ***p < 0.001, ****p < 0.0001. **Group A**, infants delivered by cesarean section and exclusively breastfed; **Group B**, infants delivered by cesarean section and fed with standard formula; **Group C**, infants delivered by cesarean section and fed with probiotic-fortified formula

*Bifidobacterium* was significantly more abundant in Group C (80.1%, 55.6–96.6%) compared to Group A (33.4%, IQR 16.1–49.3%) and Group B (27.5%, 13.8–42.0%) (*p* = 0.001)(Fig. 4A). Post-hoc analysis revealed a significant difference between Group B and Group C (*p* < 0.001), while no significant difference was observed between Groups A and B. For *Lactobacillus*, Group A exhibited the highest relative abundances (19.1%, 2.8–31.2%) compared with both Group B (4.2%, 0.9–14.3%) and Group C (0.99%, 0.3–1.6%) (Fig. 4B). All pairwise comparisons between groups were statistically significant (Group A vs. B, A vs. C, and B vs. C; all *p* < 0.001).

## Discussion

In this study of infants born by cesarean delivery, we observed distinct gut microbiota composition based on feeding modality during the early postnatal period. While probiotic-fortified formula was associated with a higher abundance of *Bifidobacterium*, it was accompanied by reduced overall microbial diversity compared to exclusive breastfeeding. The incidence of AD was similarly low in the breastfed and probiotic-supplemented groups, whereas the standard formula group showed a markedly higher incidence, despite the lack of statistically significant differences.

These findings are consistent with previous studies in which vaginal delivery and breastfeeding promoted greater microbial diversity (Madan et al., 2016). Although microbial diversity was reduced in the probiotic-fed cesarean group, the elevated abundance of *Bifidobacterium* suggests successful colonization of supplemented strains. Despite differences in microbial composition, the comparable incidence of AD between the breastfed and probiotic groups suggests that early probiotic fortification might help mitigate the immunological disadvantages associated with cesarean delivery.

Mode of delivery is a well-established determinant of early gut microbial composition (Mitchell et al., 2020). Vaginally delivered infants typically develop an anaerobic, acidic gut environment that favors colonization by Bifidobacteriaceae, while cesarean-delivered infants are characterized by a delayed colonization of *Bifidobacterium* species during the early postnatal period (Dierikx et al., 2022; Fukuda et al., 2011; Penders et al., 2006). Feeding mode further modulates the infant gut microbiome, and its effects can compound or mitigate delivery-mode differences (Fukuda et al., 2011). For example, infants born via cesarean section and fed formula have significantly reduced levels of *Bifidobacterium* and *Bacteroides* compared with vaginally delivered, breastfed infants (Davis et al., 2022b). In our cohort, the probiotic-fortified formula group (Group C) exhibited *Bifidobacterium* abundance of 80.1%, higher than previously reported levels in cesarean-delivered, formula-fed infants (32.1%) and even vaginally delivered, breastfed infants (47.1%) at similar time points (Ma et al., 2022). This suggests that probiotic fortification can effectively compensate for the deficit typically associated with cesarean birth. The probiotic-fortified formula used in Group C was specifically designed to enhance *Bifidobacterium* colonization, and our findings support its potential clinical relevance by demonstrating comparable AD incidence despite cesarean-associated microbial disadvantage.

Early colonization of *Bifidobacterium* plays an important role in immune development. Clinical trials have shown that supplementation significantly increases *Bifidobacterium* abundance, decreases colonization by potential pathogens like *Escherichia/Shigella*, improves microbial diversity, and subsequently reduces AD development (Varela-Trinidad et al., 2022). In our study, despite reduced microbial diversity, the probiotic-fortified group exhibited a *Bifidobacterium*-dominant profile and a comparable incidence of AD to that of exclusively breastfed infants. These findings support the potential immunologic benefits of early probiotic intervention in cesarean-delivered infants.

Mechanistically, *Bifidobacterium* supports immune regulation by enhancing Treg responses, preserving gut barrier integrity, modulating dendritic cell and macrophage activity, and suppressing Th2/Th17 pathways (Gavzy et al., 2023; Guo et al., 2017; Verma et al., 2018). *B. infantis* has been shown to reduce Th2/Th17 cytokine production and enhance Th1 polarization in infants (Henrick et al., 2021). These findings are supported by animal models demonstrating JAK-STAT-mediated Th1/Th2 regulation (Ding et al., 2024; Fang et al., 2022). Consistent with these mechanisms, our previous murine study demonstrated that oral administration of *B. longum* improved skin barrier function and reduced Th2-driven inflammation (Kim et al., 2022). In treated mice, the expression of key epidermal proteins, including filaggrin, loricrin, keratin-10 , and desmoglein-1, was significantly increased, while dermatitis scores, transepidermal water loss, epidermal thickness, serum IgE levels, and IL-5 expression were significantly reduced (Kim et al., 2022).

The present study has several limitations. First, we applied two different sequencing methodologies based on the time of sample collection: 16S rRNA V3–V4 region sequencing for samples collected before 2023 and full-length rRNA operon sequencing for those collected thereafter. Although previous studies have reported high concordance between these methods at the genus level, minor differences in taxonomic resolution may exist. In addition, β-diversity analysis could not be conducted due to technical challenges in integrating data from the two sequencing platforms. Second, although the proportion of infants receiving additional probiotic supplements did not significantly differ among groups, we did not account for variability in formulation, dosage, or duration, which may influence gut microbiota composition. Therefore, caution is warranted when interpreting the observed microbial differences. Third, our study did not include a comparison group of vaginally delivered infants fed probiotic-supplemented formula, limiting our ability to assess the potential interactive effects of delivery mode and feeding type. This restricts the generalizability of our findings. In addition, as this study is based on observational cohort data, the findings should be interpreted as associations rather than causal relationships. Further, detailed information on the dosage, duration, and specific strains of additional probiotics was unavailable, which might have influenced gut microbiota composition and should be considered as a limitation of this study. Nonetheless, our findings revealed that probiotic-fortified formula feeding was associated with an increased abundance of *Bifidobacterium*, supporting its potential to modulate early gut microbiota composition. Future studies should incorporate standardized sequencing protocols and broader population groups to more comprehensively evaluate the clinical implications of early probiotic intervention.

## Conclusion

Among cesarean-delivered infants at 6 months of age, the incidence of AD in those fed with probiotic-fortified formula was comparable to that of exclusively breastfed infants. The probiotic-fortified group showed an increase in *Bifidobacterium* abundance, suggesting that probiotic supplementation via formula may help shape the early gut microbiota. Long-term studies are needed to determine whether early microbiota via probiotic-fortified feeding can influence immune tolerance and modulate skin inflammation via the diet-skin-gut axis.

## Data Availability

The data are not publicly available due to privacy and ethical restrictions.
